# Supplementary material for: Affinity of rhodopsin to raft enables the aligned oligomer formation from dimers: Coarse-grained molecular dynamics simulation of disk membranes
Source: PLoS One. 2020 Feb 7;15(2):e0226123. doi: 10.1371/journal.pone.0226123 (PMC7006936; doi:10.1371/journal.pone.0226123)
Supplement: S1 Fig — (a) Relationship between H1/H8 dimer structure obtained by X-ray crystal structure analysis (ribbon model) seen from H8 side (PDB ID: 3CAP) and particle positions of the 2-D model of H1/H8 dimer (black rings). (b) Constructions of 2-D models of H1/H8 dimer consisting of 16 particles. Each particle was named 1st through 16-th and assumed to connect with nearby particles by springs where the natural length of the spring connecting the i-th and j-th particles (dijRh; S1 Table) was given to maintain the basic structure of Rh-dimer. The 8th and 16th particles (yellow) indicate the particles corresponding to the palmitoylated areas of H8 and 3rd and 11th particles (purple) indicate the Rh-Rh interface particles. Blue and purple curves indicate examples of connection between the 4th and others particles and the 7th and other particles. The dijRh for each pair of basic structures of Rh-dimers are shown in S1 Table. (c) Relationship between the expected H4/H5 dimer structure and particle positions of the 2-D model of the H4/H5 dimer. The expected H4/H5 dimer structure (ribbon model) was artificially constructed from the X-ray crystal structure of the H1/H8 dimer. The positions on the left and right Rh-monomers in H1/H8 dimer were interchanged. (d) Constructions of the 2-D models of the H4/H5 dimer consisting of 16 particles with the natural length of the spring connecting i-th and j-th particles (dijRh; S2 Table). (DOCX) [file pone.0226123.s001.docx]

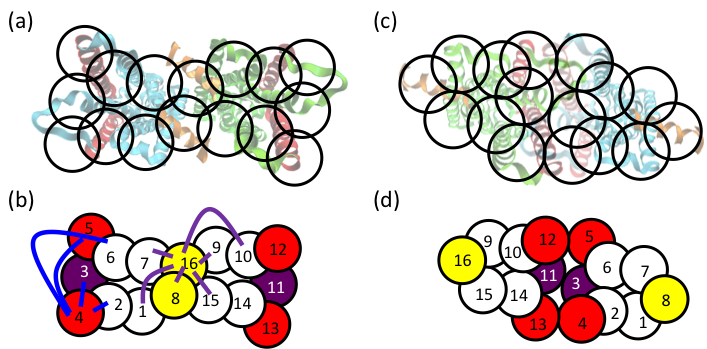


**S1 Fig. Detailed construction of a two-dimensional (2-D) elastic network model of rhodopsin (Rh) H1/H8 and H4/H5 dimers.**
