# Supplementary figures and images for: Affinity of rhodopsin to raft enables the aligned oligomer formation from dimers: Coarse-grained molecular dynamics simulation of disk membranes
Source: PLoS One. 2020 Feb 7;15(2):e0226123. doi: 10.1371/journal.pone.0226123 (PMC7006936; doi:10.1371/journal.pone.0226123)

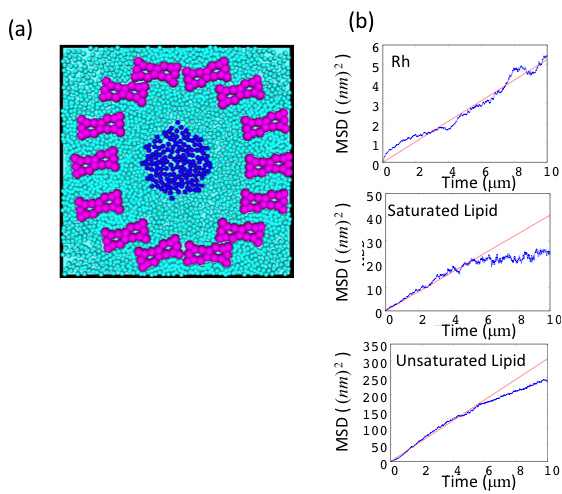


**S2 Fig. Evaluations of diffusion coefficients of rhodopsin (Rh) and lipids.**

Supplement: S2 Fig — (a) Typical initial configuration of particles to measure the diffusion coefficients of saturated lipids in a saturated lipid domain, unsaturated lipids in an unsaturated lipid domain, and Rh-dimers in an unsaturated lipid domain. The number of particles and the area fraction of two types of lipids and Rh-dimers were assumed to be the same as other simulations as in Fig 2 although saturated lipids accumulated artificially. The model with raftophobic H1/H8 dimers (εraft = 0 was assumed) was simulated in order to estimate diffusion properties of each molecule. (b) Mean square displacement (MSD (nm2)) of (Upper) Rh-dimer in unsaturated lipid domain, (Middle) unsaturated lipids in unsaturated lipid domain, and (Lower) saturated lipid in saturated lipid domain obtained by simulations from initial configurations as mentioned in (a). Red lines indicate the fitting line to infer diffusion constants. (DOCX) [file pone.0226123.s002.docx]

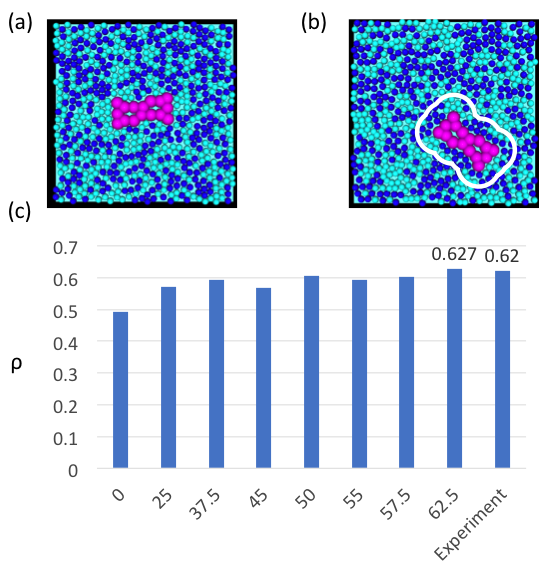


**S3 Fig. Estimation of raftophilicity of rhodopsin (Rh)-dimers.**

Supplement: S3 Fig — (a) Typical initial state of the simulations to determine the appropriate value of εraft. One Rh was inserted with the saturated and unsaturated lipids being randomly distributed. A total of 340 saturated lipids and 370 unsaturated lipids were confined to a 24 μm × 24 μm square box with periodic boundary conditions. Since unsaturated particles can overlap with each other more easily than saturated lipids, the area fraction of saturated and unsaturated lipids obtained were approximately the same, even though the absolute number of the two types of lipids differed. (b) Typical snapshot of model simulations from the abovementioned initial conditions. The vicinity of the Rh area was defined by the area surrounded by the white curve, which was closer than 2.4 nm from each particle. (c) Ratio of saturated lipids around the Rh-dimer defined by ρ=NsNs+Nu, predicted by experimental findings [Y. Tanimoto, submitted] and that of various raftophilicity εraftkBT, where Ns, Nu indicated the number of saturated and unsaturated lipids in vicinity of Rh. In the case of εraftkBT∼62.5, the ρ was 0.627, which was close to the expected experimental value of approximately 0.62 [Y. Tanimoto, submitted]. (DOCX) [file pone.0226123.s003.docx]

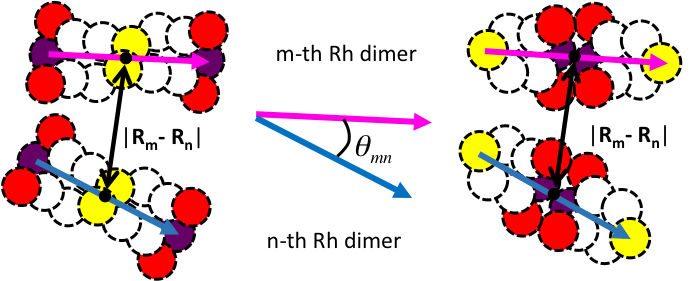


**S4 Fig. Illustration of the angle of orientations of two rhodopsin (Rh)-dimers.**

Supplement: S4 Fig — The distance and angle between the m-th and n-th Rh-dimers are shown (Left: H1/H8 dimer, Right: H4/H5 dimer), |Rm−Rn| (black arrow) and θmn. The value for cos(θmn) was determined from the inner product between the vector from one Rh-Rh interface particle to another of the m-th Rh-dimer (purple arrow) and that of n-th Rh-dimer (blue arrow). (DOCX) [file pone.0226123.s004.docx]

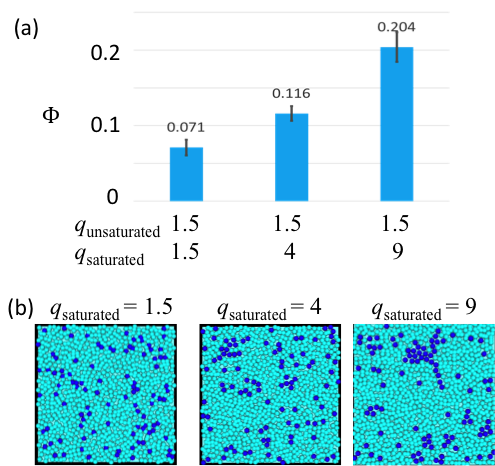


**S5 Fig. Evaluation of phase separation of saturated and unsaturated lipids.**

Supplement: S5 Fig — (a) The ratio of accumulated saturated lipids (ϕ) as a function of qi of saturated lipids, qsaturated, when qi of unsaturated lipids, qsaturated = 1.5. The value of ϕ was estimated by the equation ϕ=<NsNs+Nu>t where Ns and Nu indicate the number of saturated and unsaturated of lipids, respectively, within 1.2 nm from each saturated lipid. The area fractions of saturated lipid (blue particles) and unsaturated lipid (cyan particles) was set at approximately 8% (80 particles) and 92% (920 particles), respectively. When qsaturated = qunsaturated = 1.5, ϕ was estimated to be 0.08 as the ratio of saturated was 8%. In cases of qsaturated = 4 and 9, ϕ exhibited significantly larger values than that in the case of qsaturated = 1.5. This suggested that due to their rigidity, saturated lipids tended to form domains that may correspond to “raft lipid domain”. (b) Snapshots of saturated and unsaturated lipid configurations at qi= 1.5,4,9. The greater the qi value resulted in increased amounts of raft lipid domain that could be observed. (DOCX) [file pone.0226123.s005.docx]
