# Supplementary material for: Affinity of rhodopsin to raft enables the aligned oligomer formation from dimers: Coarse-grained molecular dynamics simulation of disk membranes
Source: PLoS One. 2020 Feb 7;15(2):e0226123. doi: 10.1371/journal.pone.0226123 (PMC7006936; doi:10.1371/journal.pone.0226123)
Supplement: S6 Fig — To evaluate saturated lipid domain-size dependent stability of inter-Rh-dimer contact, the saturated lipid model was simulated with only two Rh-dimers, several different numbers of saturated lipids (Ns = 0–7), and unsaturated lipids. (a) Typical initial configurations of molecules where saturated lipids were placed between parallel two Rh-dimers. The additional space was filled with unsaturated lipids. The distance between two centers of Rh-dimer was set at 4.8 nm. (b) Typical simulation snapshots at time (t) = 5 μs. (DOCX) [file pone.0226123.s006.docx]

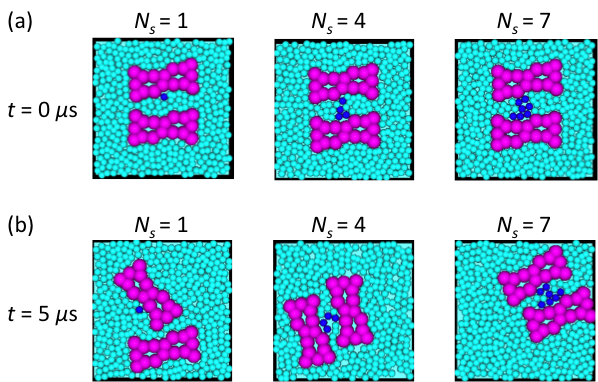


**S6 Fig. Simulation setup and results to evaluate the stability of inter rhodopsin (Rh)-dimer contact through saturated lipids.**
