# Supplementary material for: Affinity of rhodopsin to raft enables the aligned oligomer formation from dimers: Coarse-grained molecular dynamics simulation of disk membranes
Source: PLoS One. 2020 Feb 7;15(2):e0226123. doi: 10.1371/journal.pone.0226123 (PMC7006936; doi:10.1371/journal.pone.0226123)
Supplement: S1 Table — (DOCX) [file pone.0226123.s007.docx]

**S1 Table.** *d_ij_* of connected particle pairs of H1/H8 dimer model.

| Connections | *d_ij_* (nm) | Connections | *d_ij_* (nm) | Connections | *d_ij_* (nm) |
| --- | --- | --- | --- | --- | --- |
| 1-2, 9-10 | 1.28 | 1-3, 9-11 | 2.36 | 7-16, 8-15 | 1.28 |
| 2-3, 10-11 | 1.28 | 2-4, 10-12 | 1.12 | 7-15 | 2.544 |
| 3-4, 11-12 | 1.464 | 3-5,11-13 | 1.28 | 8-16 | 1.08 |
| 4-5, 12-13 | 2.744 | 4-6, 12-14 | 2.56 |  |  |
| 5-6, 13-14 | 0.976 | 5-7, 13-15 | 2.216 |  |  |
| 6-7, 14-15 | 1.28 | 6-8, 14-16 | 2.432 |  |  |
| 7-8, 15-16 | 1.28 | 1-7, 9-15 | 1.808 |  |  |
| 1-8, 9-16 | 1.48 | 2-8, 10-16 | 2.544 |  |  |
