# Supplementary material for: Affinity of rhodopsin to raft enables the aligned oligomer formation from dimers: Coarse-grained molecular dynamics simulation of disk membranes
Source: PLoS One. 2020 Feb 7;15(2):e0226123. doi: 10.1371/journal.pone.0226123 (PMC7006936; doi:10.1371/journal.pone.0226123)
Supplement: S3 Table — The findings were obtained by model simulation and published experimental results [1–4]. Diffusion coefficients of molecules in the current simulations were estimated by fitting of the mean square displacement (S2B Fig). (DOCX) [file pone.0226123.s009.docx]

**S3 Table.** Diffusion coefficients of Rh-dimer in unsaturated lipid domains, unsaturated lipids in unsaturated lipid domains, and saturated lipid in saturated lipid domains. The findings were obtained by model simulation and published experimental results [1–4]. Diffusion coefficients of molecules in the current simulations were estimated by fitting of the mean square displacement (S2b Fig).

| Particle type | Experiment [µm^2^/s] | Simulation  [µm^2^/s] |
| --- | --- | --- |
| Rhodopsin | 0.36 [1,2] | 0.52 |
| Saturated lipid | 5.0 [3,4] | 3.09 |
| Unsaturated lipid | 10.0 [3,4] | 27.05 |

1. Tanimoto Y, Okada K, Hayashi F, Morigaki K. Evaluating the raftophilicity of rhodopsin photoreceptor in a patterned model membrane. Biophys J. 2015; 109: 2307–2316. doi:10.1016/j.bpj.2015.10.015.

2. Hayashi F, Saito N, Tanimoto Y, Okada K, Morigaki K, Seno K, et al. Raftophilic rhodopsin-clusters offer stochastic platforms for G protein signalling in retinal discs. Commun Biol. 2019; 2: 1–12. doi:10.1038/s42003-019-0459-6.

3. Almeida PFF, Vaz WLC, Thompson TE. Lipid diffusion, free area, and molecular dynamics simulations. Biophys J. 2005; 88: 4434–4438. doi:10.1529/biophysj.105.059766.

4. Scherfeld D, Kahya N, Schwille P. Lipid dynamics and domain rormation in model membranes composed of ternary mixtures of unsaturated and saturated phosphatidylcholines and cholesterol. Biophys J. 2003; 85: 3758–3768. doi:10.1016/S0006-3495(03)74791-9.
