# Supplementary material for: Affinity of rhodopsin to raft enables the aligned oligomer formation from dimers: Coarse-grained molecular dynamics simulation of disk membranes
Source: PLoS One. 2020 Feb 7;15(2):e0226123. doi: 10.1371/journal.pone.0226123 (PMC7006936; doi:10.1371/journal.pone.0226123)
Supplement: S1 File — (DOCX) [file pone.0226123.s010.docx]

**S1 Text. Computation time for simulations of the present study.**

The present simulation results were mainly obtained by parallel computing using 3 CPUs (Intel Core i9-7900X, 3.30 GHz) with Open MP. The computation time for the simulation required to give one 200μs time course (as in Fig. 2) was around 10 days.
